# Chemical Composition and Glycemic Index of Gluten-Free Bread Commercialized in Brazil

**DOI:** 10.3390/nu12082234

**Published:** 2020-07-27

**Authors:** Bernardo Romão, Raquel Braz Assunção Botelho, Ernandes Rodrigues Alencar, Vera Sônia Nunes da Silva, Maria Teresa Bertoldo Pacheco, Renata Puppin Zandonadi

**Affiliations:** 1 Department of Nutrition, University of Brasília, 70910-900 Brasília, Federal District, Brazil; raquelbotelho@unb.br; 2 Agronomy and Veterinary Faculty, University of Brasília, 70910-900 Brasília, Federal District, Brazil; ernandesalencar@gmail.com; 3 Institute of Food Technology, 13070-178 Campinas, São Paulo, Brazil; vera.silva@ital.sp.gov.br (V.S.N.d.S.); mtb@ital.sp.gov.br (M.T.B.P.)

**Keywords:** gluten-free bread, glycemic index, nutritional quality, gluten-related disorders

## Abstract

Nowadays, the world is experiencing an increased demand for gluten-free products associated with the high prevalence of gluten-related disorders (GRD). As this market thrives, bread stands out as the most demanded and consumed product, highlighting the need to evaluate its nutritional quality. In this sense, this cross-sectional study aimed to assess the ingredients, chemical composition, and glycemic index of gluten-free bread (GFB) commercialized nationwide in Brazil. The labels were analyzed according to their ingredients and composition. In addition, centesimal composition analysis (moisture, carbohydrates, protein, ash, lipid, and fiber) was performed using the official methods, as well as the in vivo glycemic index. Significant differences between manufacturing lots of each brand and between different gluten-free bread samples were found. There is a mismatch from what is stipulated by the Brazilian legislation between analytical data and the data described on sample labels. Samples showed predominantly refined starch and high glycemic index profile. Most samples (75%, *n* = 6) of the non-whole gluten-free bread (*n* = 8) presented high glycemic index (GI). From four whole food/multigrain gluten-free bread samples, three of them (75%) presented medium GI. However, two samples presented GI near the value to be considered high. The results suggest a lack of production control, impairing the gluten-free nutritional label as a reliable information source and tool for dietary control. Therefore, it is necessary to standardize the process of gluten-free bread production and labeling, as well as to improve the nutritional characteristics of these products, aiming the give accurate information to consumers and provide a healthier product beyond the sensory characteristics.

## 1. Introduction

Worldwide, there has been an increase in demand for gluten-free products associated with the high prevalence of gluten-related disorders (GRDs) [1]. Among the GRDs, the far more widely known disorders are celiac disease (CD), wheat allergy (WA), and non-celiac gluten sensitivity (NCGS) [1,2,3]. Although studies are looking for new treatment alternatives for GRDs, the total exclusion of gluten and/or wheat from the diet is still the only safe treatment [4,5]. In addition to individuals with DRGs, their family members tend to consume gluten-free products to support their treatment and avoid cross-contamination of food. Some individuals without GRD have adhered to gluten-free diets (GFDs). They believe in the potential health benefits of GFDs, despite the lack of scientific evidence related to such practice [6,7,8,9,10]. Therefore, the estimation is that about 10% of the world population (with or without GRD) has adopted a GFD [2,3,11,12].

At the same time, with the increasing number of people who adhere to GFD, the market for these products also grows, but there are still doubts about their nutritional quality [13]. Starches and flours from rice, corn, and potatoes with lower micronutrients, protein, dietary fiber content, and a high glycemic index tend to be used to produce well-accepted products [7,11,14].

Although responses may suffer influence from individual factors such as microbiome and genetic factors, the glycemic index (GI) is a useful tool for the nutritional quality assessment of foods [15,16]. Studies have shown that foods with high and moderate (close to the upper limit) glycemic indexes are less healthy options. In addition, they are associated with the early development of non-communicable diseases (NCD) such as diabetes and overweight [17]. Furthermore, in the case of individuals adhering to a GFD, the ingredients commonly used in the formulations have high GIs, and the risk of developing NCD may be higher [18,19,20].

A product that tends to present high GI and is among the most consumed and desired gluten-free products by DRGs individuals is bread [21,22]. Bread constitutes one of the most consumed products in Brazil (34 kg per capita/year). Its technological and sensory characteristics mainly rely on the presence of gluten, hence the difficulty of developing satisfactory gluten-free versions [23,24,25,26].

With gluten withdrawal, refined ingredients, with high glycemic indexes, are used to achieve desirable sensory characteristics, not prioritizing the nutritional aspects [14,18,24]. This tendency has been verified in most studies regarding gluten-free bread using higher fat and carbohydrates contents and presenting high GIs [14,27,28]. Gluten-free bread tends to be high in lipids and additives (mainly gums and fibers) in addition to starches. There is still a lack of knowledge about the ingredients in gluten-free bread formulation and their effect on the gluten-free bread GI. Based on this information, the high GI profile within gluten-free bread and the potential nutritional shortcomings regarding its extended consumption, this study aimed to evaluate the chemical composition and GI of gluten-free bread commercialized in the Brazilian market.

## 2. Materials and Methods

This cross-sectional quantitative research was conducted in four steps: (i) sample mapping and selection; (ii) determination of chemical composition; (iii) in vivo GI gauging; (iv) statistical analysis.

### 2.1. Sample Mapping and Selection

Sample mapping was conducted between August 2018 and January 2019. To map the samples, researchers looked for gluten-free bread in supermarket chains with national coverage (*n* = 4) and in alternative food stores with regional coverage (*n* = 2) and local coverage (Federal District, Brazil) (*n* = 24). After evaluating all the commercialized types of bread, we contacted the manufacturers of each bread brand to confirm the scope of distribution. In addition, we questioned them about the methods used in the development of the nutritional label. We searched the Brazilian e-market for gluten-free bread; however, availability was the same as physical stores for room temperature samples.

The researchers only included the brands commercialized in all the five Brazilian regions. In total, 12 samples of gluten-free bread were analyzed (8 of them were the traditional gluten-free bread—GFB; and 4 of them were whole/multigrain versions samples—WGFB). The study had two international samples and ten Brazilian samples.

For each type of bread, products from three different manufacturing lots were purchased, for a more reliable composition of these products [29].

### 2.2. Chemical Composition

All the analyses were performed in triplicate. Total moisture was determined following the analytical standards of the Adolfo Lutz Institute [30], in which 30 g of each gluten-free bread sample was inserted in a melting pot (previously dried in a drying oven at 105 °C, for 24 h, and placed to cool in a glass desiccator—Pyrex^®^ (Corning Inc., Corning, NY, USA) for 45 min). The samples were dried in a drying oven at 105 °C, for 24 h and placed to cool in a glass desiccator—Pyrex^®^, USA, for 45 min. The melting pot with each sample was weighed on an analytical scale (BOSCH^®^, Gerlingen, Germany—SAE 200). The moisture content was obtained by the formula: % Moisture = 100 − {[(Melting pot weight + Dried sample 105 °C) − Melting pot weight] × 100} [30]. Ashes [31], fat [32], protein [33]**,** and dietary fiber [34] for each manufacturing gluten-free bread lot were also determined. In total, nine samples for each type of bread were analyzed. Available carbohydrate content was obtained by the difference from the investigated composition, including dietary fiber.

### 2.3. In Vivo GI Gauging

The in vivo GI determination was performed following Food and Agriculture Organization’s (FAO) 1998 protocol and the recommendations from Brouns et al. [35,36]. Volunteers were recruited online. The exclusion criteria were: individuals using medications, smokers, with disorders within glucose metabolism, and the presence of GRD. This phase was performed at the University of Brasilia, between August and December of 2019. Data collection started at 7 a.m., with a minimum interval of 3 days between each gauging and a maximum of two weeks. Ten healthy male individuals participated in this phase, with the following mean characteristics: 23.67 ± 2.00 years, 72.84 ± 8.12 kg, 1.76 ± 0.06 m, body mass index (BMI) of 23.50 ± 2.90 kg/m^2^, body fat percentage of 19.33 ± 6.61 and lean mass percentage of 39.78 ± 4.67.

Volunteers fasted for a minimum of ten and a maximum of twelve hours before blood analysis. Accu-check^®^ (Roche, São Paulo, Brazil) kit for glucose monitoring was used in order to collect finger-pricked capillary blood. Capillary blood glucose was collected on an empty stomach (t = 0) followed by gluten-free bread sample consumption with a total of 50 g of available carbohydrates (provision of 250 mL of water). After each bread intake in each analysis day, researchers evaluated blood glucose after 15, 30, 45, 60, 90, and 120 min. White bread was used as a standard food and twice evaluated in different weeks [35]. Each gluten-free bread sample was analyzed on different days, respecting a minimum interval of three days and a maximum of two weeks between different bread samples. Volunteers were informed to maintain their regular dietary habits and to repeat their usual last meal of the day before each test day [36].

For blood glucose analysis, the incremental area under the curve (_i_AUC) was calculated by geometrically applying the trapezoid rule for each individual and each sample (twice for the standard food), and the results were averaged into a mean _i_AUC value. The area under the baseline (fasting) was not considered. Given that white wheat bread was used as the standard food, GI values were multiplied by 0.7 to obtain values with glucose as the standard food (GI = 100) [37]. The following formula was applied to calculate the glycemic index: GI = [(_i_AUC GFB/_i_AUC Standard food) × 100]. Then, the GI was classified as high (>70), medium (56–69), and low (<55) [36,37].

The study was approved at the University of Brasilia, Faculty of Health Sciences ethics committee (protocol number CAAE 09227718.8.0000.0030).

### 2.4. Statistical Analysis

Repeated measures analysis of variance (ANOVA) and Tukey test (95%, *p* < 0.05) were performed to evaluate differences in GI and chemical composition from different gluten-free bread and different manufacturing lots from the same brand. Brazilian law stipulates a tolerance value of 20% for discrepancies between the chemical composition from the laboratory and the nutritional label. Therefore, percentage differences between the mean laboratory values and those described by the labels were calculated [29].

Chemical composition values were expressed by the mean of 3 replicates of 3 manufacturing lots (*n* = 9) ± standard deviation (SD). Word clouds were generated for samples classified as high and medium GI, based on gluten-free bread ingredients frequency in the labels, given that higher frequencies would mean more extensive graphical representations (Supplementary file—Figure S2). Common ingredients in any type of commercialized bread formulations were not included, these being: sugar, salt, yeast, and calcium propionate. The software Xlstat^®^ (Addinsoft, Paris, France, 2018) was used to perform all statistical analyses.

## 3. Results

The ingredients used in Brazilian commercialized gluten-free bread brands and their respective values for chemical composition and GI are available in Table 1 and Table 2. Corn, cassava, potato, and rice starches/flours are commonly used in gluten-free bread formulations and they were present in 83.3% (*n* = 10), 58.3% (*n* = 7), 16.6% (*n* = 2), and 16.6% (*n* = 2) of the samples, respectively (Table 1).

Unspecified modified starch was used in 33.3% (*n* = 4) of the samples, two samples of GFB (2, 3), and two of WGFB (2, 3). All of the gluten-free bread samples combined a minimum of two starch sources, with the combination of cassava and corn starches the most frequent one, and rice flour being the third most used ingredient. The carbohydrate values ranged from 33.32 to 55.92 g/100 g among the samples.

Xanthan gum was used as a gluten substitute in all the gluten-free bread formulations. Its graphical representation in the word cloud was the largest one in both word clouds for gluten-free bread classified as medium and high GI (Table 1 and Table 2). Hydroxypropyl-methylcellulose (HPMC), another hydrocolloid, was used in 41.6% (*n* = 5) of gluten-free bread samples and predominantly in samples classified as medium GI (Supplementary file—Figure S2). Guar gum was present in 16.66% (*n* = 2) (Table 1 and Figure S2).

Ingredients such as egg (25%, *n* = 3), egg powder (8.33%, *n* = 1), soy protein (16.66%, *n* = 2), soy flour (58.33%, *n* = 7), and soy extract (16.66%, *n* = 2) were used as primary protein sources. GFB samples mainly presented soy flour as their main protein source (41.66%, *n* = 5), while WGFB presented different primary sources: soy protein (WGFB 1), egg powder (WGFB 2), soy extract (WGFB 3) and soy flour (WGFB 4). Protein values ranged from 2.22 to 8.34 g/100 g.

All formulations used one type of lipid source—either soy oil (50%, *n* = 6), sunflower oil (25%, *n* = 3), corn oil (16.66%, *n* = 2), or palm fat (16.66%, *n* = 2). Fat content varied between 0.75 and 4.75 g/100 g, whereas higher values were from the formulation that implemented corn oil as the main fat source (GFB 2). Dietary fiber sources were present in all GFB formulations, mainly flaxseed in 41.66% (*n* = 5) of the samples, psyllium (16.66%; *n* = 2), chia seeds and quinoa (16.66%; *n* = 2). Chia and quinoa were only present in WGFB. Values ranged from 6.79% up to 17.21%, whereas formulations with psyllium presented higher values (GFB 1 and WGFB 1; 17.21% and 16.26%, respectively).

Most of the GFB samples (58%, *n* = 7) presented a high GI profile, and 42% (*n* = 5) presented medium GI. However, when analyzed separately, 75% (*n* = 6) of GFB (analogs to white bread) were classified as high, and only one sample (25%) of WGFB presented the same classification. GI values ranged from 64.00 to 79.94 in GFB, and from 61.46 to 75.40 in WGFB (Table 2). Mean _i_AUC for standard food (white bread) was 577.38 ± 59.97. In the supplementary file, Figure S1 presents the mean glycemia values for all analyzed GFB and Table S1, the mean glycemia values in Mmol/L for GFB samples.

Brazilian legislation for food label development states that chemical composition can be determined by analytical methods or based on food composition tables [29]. The researchers contacted the GFB manufacturer’s customer service to evaluate which method was used to compose the label of the products. However, none of them explained to researchers the method used to inform the chemical composition presented in the labels. Within all samples, different lots from the same product presented significant differences in the chemical composition (Table 3). Only total fat values among lots of GFB2 and protein content of the WGFB4 lots were not significantly different. In addition, differences between the analyzed chemical composition and values reported on the nutritional label were found (Table 3). Brazilian legislation stipulates 20% as the tolerance value [29] for labeling information.

In relation to the mean chemical composition of the analyzed gluten-free bread and those described on the labels (Table 3), 25% of the samples (*n* = 3) presented differences between labels and analysis beyond the tolerance value for the carbohydrates. For fat and protein, 91.6% (*n* = 11) of the samples were different and above the tolerance value, and 100% (*n* = 12) for dietary fiber (Table 3). The difference between the chemical analysis and the information on labels (in percentages) ranged from 20.09% to 34% for carbohydrates, 26.27% to 89.19% for protein, 28.44% to 300% for fat, and 38.5% to 86.18% for dietary fiber.

## 4. Discussion

Although a few studies have already discussed some GFB ingredients and nutritional label information, this study is the first one providing analytical data regarding the chemical composition, and the in vivo GI of GFB commercialized in Brazil [38,39,40].

In traditional wheat bread, both wheat-starch and gluten are responsible for the desirable technological characteristics, such as texture, volume, color, crust, and crumb structure [41]. In gluten-free bread production, different combinations of starches and gluten-substitutes must be explored [24,42].

Starch is the main compound in bread, contributing mainly to the texture and structure [43,44,45]. The inefficiency of using a single starch source in the GFB production leads to a combination of starches in order to improve the technological and sensory characteristics of the products [25], as shown in Table 1.

Corn, potato, cassava, and rice flours/starches were the main ingredients in the analyzed GFB samples, confirming the pattern evidenced in studies about the main ingredients used in Brazilian gluten-free bread [40]. Cassava starch, corn starch, and modified (non-specific) starch were the primary sources of starches in samples with a high GI (Supplementary file—Figure S2). In the medium GI samples, corn starch was the primary source of starch (Supplementary file—Figure S2). Different from samples that presented high GI, samples with medium GI presented quinoa, chia, sunflower seed, flaxseed, psyllium, and rice flour (Supplementary file—Figure S2).

In gluten-free bread, hydrocolloids are important because they create stable structures capable of retaining growth while providing desirable crumb textures for bread [25,44]. Regarding nutritional and health aspects, xanthan and guar gums act similarly to dietary fibers, as prebiotics, improving satiety, gut functioning, and, most importantly, slowing down carbohydrates digestion, decreasing foods’ GI [46,47]. However, these gums were present in all analyzed GFB samples, mostly presenting a high GI profile, suggesting that in the context of these GFBs (samples 3–8 and 12), the implemented quantities of this class of ingredient were insufficient to exert influence on the GI. 

Similarly, this occurs with dietary fiber, protein, and fats, which are nutritional compounds that form heavy molecular complexes that slow down carbohydrates’ digestion, and supposedly, reduces the foods’ GI [20]. Heterogenous results were found within GFB samples. The increased amounts of these nutrients in the production did not necessarily result in a proportional reduction in GIs in the final product.

In comparison with other analyzed GFB samples, GFB 1 showed higher values for dietary fiber (Table 2), mainly because of the use of psyllium (*Plantago ovata*). Psyllium is a type of viscous mucilage regularly used to replace gluten and to reduce foods’ GI [48,49]. At the same time, GFB 1 showed lower values for available carbohydrates, which might have contributed to the medium GI. However, GFB 2 was also classified as medium GI and presented less than half of the amount of fiber compared to GFB 1. In addition, GFB 2 was not significantly different from 41.66% of the samples classified as high GI. However, surprisingly, its fat content was the highest among all samples (4.36 g/100 g), suggesting that the interaction of both fat and fiber might have influenced the sample’s GI [20]. Other samples (GFB 3–8) presented values for dietary fiber above 6 g/100 g, classified by the Brazilian legislation as a “fiber-rich” food. Lipid values ranged from 2 to 3.9 g/100 g. Therefore, besides the lipids and dietary fiber amounts, the nature of the implemented ingredients might exert a direct influence on the GI. In the context of the analyzed GFB, the amount of refined starches and sugars in GFB formulations (Table 1) might have prevailed over other ingredients’ capacity to influence the glycemic response, therefore affecting the GI profile.

Naturally, given their refined nature, the starches and flours in GFB and WGFB formulations tend to present high GIs, such as 70 for corn and 86 for cassava starches [37,50]. Considering that these starches were mainly used in GFB and WGFB, and represent most of the sample’s mass and carbohydrate values, this class of ingredient might be the primary influence on the obtained GI. Different samples of commercialized GFB around the world showed different profiles regarding GI [19,24,51]. Commercial brands sold in Italy showed high GI (100) in formulations where potato, rice, and cassava starches were implemented associated with xanthan gum as a gluten replacer, similar to the results of this present study [51]. In Iran, four GFB brands used rice as their primary starch source added with xanthan and guar gums as gluten replacements, showing a high GI profile (86, 89, 76, 74) [24]. Brands with medium GIs implemented the similar starch sources but with different gluten replacers such as apple fiber (GI = 51), lupin protein (GI = 62), and sourdough (GI = 63), suggesting that the use of these ingredients might be useful to reduce GI [19].

Among the alternatives to decrease bread GI in multiple grain-based formulations, the addition of nuts and whole seeds/flours may be a good option [18,42,48]. Gluten-free bread enriched with such ingredients tends to present higher concentrations of dietary fiber and protein [6,14]. Probably, the addition of whole-grain flours and seeds/nuts exerts influence on the technological characteristics regarding the dough’s growth, texture, and flavor, consequently impacting on the acceptance; therefore, the use of these ingredients without sensory impairing is limited [6,41].

WGFBs containing whole grains, pseudocereals, and seeds presented higher amounts of dietary fiber, and consequently lower GIs in comparison with GFB. Therefore, most of the WGFBs (75%) presented medium GI values. However, it is essential to mention that some brands presented the GFB and WGFB version. GFB 1 and WGFB 1 have similar ingredients, presenting rice flour in their composition. The difference among them is the use of flaxseed, sunflower seeds, citrus fiber (instead of carbon fiber), and caramelized sugar (as the last ingredient) in the WGFB. Although there are not criteria for classifying a cereal-based product as a whole product in Brazil, theoretically, whole grains and their by-products are foods in their most natural or least processed form [52]. Traditionally, they contain more fiber and minerals than the refined versions. The WGFB1 sample presented a similar amount of dietary fiber and a lesser amount of ashes than GFB 1.

Comparing GFB 2 and WGFB 2, the main ingredients are also similar (Table 1). However, WGFB 2 presents a different type of starch (as the second ingredient), and the addition of sunflower seeds, flaxseed meal, quinoa, chia, emulsifiers, and mono and diglycerides of fatty acids. Despite the label information of ingredients that traditionally present high fiber content and minerals (sunflower seed, flaxseed meal, quinoa, and chia), the fiber and ashes contents are similar among the samples, as well as their GI (Table 1 and Table 2). This is probably because the implemented quantities were not enough to express differences in the samples’ chemical composition, or the label information does not reflect the real ingredients used in one of those samples. Only the protein content in GFB 2 is lower than WGFB 2. Unlike the other brands, WGFB 3 had the addition of sunflower seed, quinoa, golden flaxseed, brown flaxseed, chia, and millet, lowering the GI. These ingredients increased the fiber content (Table 1 and Table 2), as expected for a whole-food version in comparison to its refined counterpart (GFB3).

Comparing GFB 4 and WGFB 4 (both with high GI), only the flaxseed flour was added in the whole-grain version, which did not affect the dietary fiber nor the ashes contents, probably because the label information does not reflect the real ingredients used in one of those samples. However, there is a difference in the order of two ingredients on the list. GFB 4 presents soy oil and soy flour, and WGFB, soy flour, and soy oil, which might have influenced the composition because, in Brazil, the order of ingredients is referred from the highest to the lowest quantity on product labels. We observed that the amount of protein in WGFB 4 is higher than GFB 4, probably due to the higher amounts of soy flour. Despite the reduction in soy oil compared to the GFB 4, the fat content was similar, probably resulting from the addition of soy and flaxseed flours, which contain lipids in their compositions.

The comparison of the traditional gluten-free bread (GFB) and whole versions of gluten-free bread (WGFB) within the same brand showed no relevant differences between the versions, and the “whole-food” claim on the label may influence the consumer’s choice with a (mistaken) feeling of acquiring a healthier product.

Multiple studies already explored and developed different alternatives to decrease gluten-free bread GI. Psyllium was used as a gluten substitute showing GFB with medium GI (64.9) [49]. Inulin-type fructans lowered GI with higher concentration. Concentrations of 0%, 4%, 8%, 10% and 12% presented GI of 65.19, 62.38, 60.48, 59.48 and 58.87, respectively [53]. In addition, different types and concentrations of implemented hydrocolloids in potato starch-based gluten-free bread lowered GI as well [47]. Gluten-free bread enriched with 2% of HPMC, CMC, xanthan gum, and apple fiber presented GIs of 58.59, 62.71, 66.25, and 65.12, respectively [47]. In both analyzed GFBs and WGFBs, HPMC was present in 100% of the samples classified as a medium GI, reinforcing its efficacy in mitigating gluten-free products GI, accordingly to the literature data [44,47,54]

Despite the implementation of similar ingredients, none of the commercialized GFBs followed the formulations described by the mentioned literature studies, thus, supporting the gap among studies regarding nutritional quality in gluten-free products and the commercial reality [55]. Probably, sensory aspects would be impaired with high amounts of fiber, and in the consumers’ habit, overall acceptance is the primary determinant for the food to be consumed [56,57].

Although the in vivo glycemic index serves as a useful tool for food quality evaluation, singular factors regarding the individuals’ glycemic response must be considered to implement this tool in the context of a dietary habit. Variabilities related to microbiomes, anthropometric measures, physical activities, and genetic factors have proven to exert influence on the glycemic response of individuals who consume the same food [16]. The validated protocol for in vivo GI gauging demands that only healthy volunteers participate. However, given the heterogenicity of the health status of different populations across the world, for results based on a strictly defined population, the glycemic response might not be related to most populations [16,35]. In the context of GRD, intestinal damage inherent to these disorders influences the digestion, probably affecting the glycemic response of food consumption and, subsequently, the GI [1]. In this study, only healthy volunteers with low variability regarding gender, age, and body composition participated, reducing the inter variability regarding their glycemic response (Table 2).

GFBs commercialized in Brazil presented divergences in the chemical composition within different lots of the same product, with higher differences (above or below) than allowed by the Brazilian legislation [29], suggesting a lack of control in the production process. These differences indicate that the composition described by the label does not reflect reality, biasing the use of the label as a tool for dietary evaluation, and impairing available information regarding GFB nutritional quality. In addition, in the context of GF products, production control is strictly necessary, because the smallest error can result in the presence of traces of gluten, thus harming the health status of any GRD patient who consumes it.

## 5. Conclusions

Despite the possibility to reduce the GI of gluten-free bread by increasing amounts of fiber, fats, and proteins in the formulation, most of the GFB (traditional and whole version) sold in Brazil presents high glycemic indexes. This is probably due to the extensive use of refined starches and sugars. No relevant differences were found among the implemented ingredients in traditional and whole-food versions of GFB. However, the fat content between two whole GFBs was significantly different and impacted GI. This could be a sign of lipids’ influence in lowering GI. The “whole-food” claim on the labels may influence the consumer´s choice with a (mistaken) feeling of acquiring a healthier product. Significant differences were found between the chemical composition of different manufacturing lots of GFB, suggesting a lack of production control and thus impairing the nutritional label as a reliable information source and tool for dietary control. Therefore, it is necessary to standardize the process of gluten-free bread production and labeling, as well as to improve the nutritional characteristics of these products, aiming to give accurate information to consumers and provide a healthier product beyond the sensory characteristics.

## Figures and Tables

**Table 1:** Ingredients described on the labels of commercial gluten-free bread.

Samples	Ingredients as Presented on Labels *
GFB 1	Water, corn starch, rice flour, vegetable fiber (psyllium), hydroxypropyl methylcellulose, sunflower oil, soy protein, biological yeast, salt, carbon fiber, sugar
GFB 2	Modified starch, corn oil, sugar, egg, salt, emulsifiers mono and diglycerides of fatty acids, hydroxypropyl methylcellulose, xanthan gum, flavoring, calcium propionate, and potassium sorbate.
GFB 3	Modified starch, starch (cassava and/or corn and/or potato), soy extract, palm fat, powdered glucose, demerara sugar, salt, hydroxypropyl methylcellulose thickeners, xanthan gum, guar gum, calcium propionate preservative, and stearoyl emulsifier 2-sodium lactyl lactate.
GFB 4	Cassava starch, corn starch, cassava, soy oil, soy flour, sugar, yeast, salt, INS 282 preservative (calcium propionate), and INS 415 stabilizer (xanthan gum)
GFB 5	Cassava starch, soy flour, egg, cornflour, corn starch, soy oil, sugar, yeast, salt, INS 282 preservative (calcium propionate), and INS 415 stabilizer (xanthan gum)
GFB 6	Cassava starch, corn starch, carrot, soy flour, soy oil, yeast, salt, INS 282 preservative (calcium propionate), and INS 415 stabilizer (xanthan gum)
GFB 7	Cassava starch, corn starch, sweet potato, soy flour, soy oil, yeast, salt, INS 282 preservative (calcium propionate), and INS 415 stabilizer (xanthan gum)
GFB 8	Cassava starch, soy flour, egg, cornflour, corn starch, soy oil, sugar, yeast, salt, INS 282 preservative (calcium propionate), and INS 415 stabilizer (xanthan gum)
WGFB 1	Water, corn starch, rice flour, vegetable fiber (psyllium), flaxseed, thickener: hydroxypropyl methylcellulose, sunflower oil, soy protein, sunflower seeds, biological yeast, salt, citrus fiber, sugar, and caramelized sugar.
WGFB 2	Modified starch, starch, corn oil, sugar, sunflower seed, egg powder, salt, flaxseed meal, quinoa, chia, hydroxypropyl methylcellulose emulsifiers, mono and diglycerides of fatty acids and xanthan gum, conservative calcium propionate and potassium sorbate
WGFB 3	Modified starch, starch (cassava and/or corn and/or potato), soy extract, sunflower seed, palm fat, powdered glucose, demerara sugar, quinoa, golden flaxseed, brown flaxseed, chia, millet, salt, thickeners hydroxypropyl methylcellulose, xanthan gum and guar gum, calcium propionate preservative, and sodium stearoyl-2-lactyl lactate emulsifier.
WGFB 4	Cassava starch, corn starch, flaxseed flour, soy flour, soy oil, organic yeast, salt, INS 415 stabilizer (xanthan gum), and INS 282 preservative (calcium propionate)

* Ingredients are listed according to their quantities, from the highest concentration to the lowest as required by the Brazilian legislation.

**Table 2:** Mean chemical composition and glycemic index for analyzed GFB.

Samples	Carbohydrates (g)	Protein (g)	Lipid (g)	Dietary Fiber (g)	Ashes (g)	Moisture (g)	Mean _i_AUC + SD	GI	Classification [37]
GFB 1	32.32 ± 1.81 ^a,^*	4.34 ± 0.57 ^a,^*	1.75 ± 0.80 ^a^	17.21 ± 1.6 ^f,^*	2.05 ± 0.38 ^b,^*	42.33 ± 2.02 ^e,^*	560.70 ± 36.25	67.97 ^b^	Medium
GFB 2	46.26± 2.41 ^c,^*	2.22 ± 0.24 ^b,^*	4.36 ± 1.34 ^b,^*	7.74 ± 1.91 ^a,^*	3.41 ± 0.16 ^a,^*	36.02 ± 1.47 ^c,^*	527.96 ± 32.88	64.00 ^a^	Medium
GFB 3	41.79 ± 1.99 ^b,^*	5.09 ± 0.23 ^c,^*	2.00 ± 0.77 ^a,^*	10.13 ± 1.05 ^d,^*	1.83 ± 0.31 ^b,^*	39.16 ± 2.73 ^d,^*	578.60 ± 34.61	70.14 ^c^	High
GFB 4	52.56 ± 2.89 ^e,^*	5.11 ± 0.40 ^c,^*	2.09± 0.65 ^a,^*	6.85 ± 0.9 ^a,^*	1.33 ± 0.13 ^a,^*	32.06 ± 2.01 ^b,^*	649.33 ± 41.82	78.72 ^e^	High
GFB 5	48.35 ± 1.34 ^c,^*	8.34 ± 0.27 ^d,^*	3.90 ± 0.64 ^b,^*	7.69 ± 1.2 ^b,^*	1.40 ± 0.23 ^a,^*	30.32 ± 1.54 ^a,^*	640.84 ± 30.11	77.69 ^e^	High
GFB 6	50.65 ± 1.28 ^d,^*	5.52 ± 0.15 ^c,^*	2.28 ± 0.27 ^a,^*	8.80 ± 1.13 ^c,^*	1.77 ± 0.08 ^a,b,^*	30.98 ± 2.39 ^a,^*	659.43 ± 54.10	79.94 ^e^	High
GFB 7	48.64 ± 1.77 ^c,^*	6.72 ± 0.15 ^e,^*	2.68 ± 0.14 ^a,^*	6.99 ± 1.16 ^a,^*	1.18 ± 0.14 ^a,^*	33.79 ± 2.60 ^b,^*	631.25 ± 44.65	76.53 ^de^	High
GFB 8	48.57 ± 1.73 ^c,^*	5.30 ± 0.14 ^c,^*	2.03 ± 0.35 ^a,^*	7.64 ± 1.04 ^b,^*	1.03 ± 0.05 ^a,^*	35.43 ± 1.37 ^c,^*	621.84 ± 33.94	75.39 ^d^	High
WGFB 1	39.66 ± 2.01 ^e,^*	4.17 ± 0.51 ^a,^*	0.50 ± 0.80 ^c,^*	16.26 ± 1.05 ^f,^*	1.31 ± 0.10 ^a,^*	38.68 ± 1.70 ^d,^*	558.16 ± 30.78	67.66 ^b^	Medium
WGFB 2	47.32 ± 1.66 ^c,^*	3.54 ± 0.63 ^a,^*	3.90 ± 0.94 ^b,^*	6.79 ± 1.07 ^a,^*	1.31 ± 0.03 ^a,^*	37.14 ± 2.28 ^d,^*	506.94 ± 46.04	61.46 ^a^	Medium
WGFB 3	40.1 ± 0.77 ^b,^*	5.87 ± 0.05 ^c,^*	3.96 ± 0.81 ^b,^*	12.56 ± 0.77 ^e,^*	1.83 ± 0.31 ^b,^*	38.68 ± 0.37 ^d,^*	571.03 ± 29.62	69.23 ^b^	Medium
WGFB 4	47.95 ± 1.42 ^c,^*	6.75 ± 0.17 ^e^	2.03 ± 0.35 ^a,^*	6.81 ± 1.31 ^a^	1.03 ± 0.05 ^a,^*	35.43 ± 1.37 ^c,^*	621.96 ± 27.16	75.40 ^d^	High

Values are expressed as the mean of triplicates of three manufacturing lots (*n* = 9) and their standard deviations. GFB = gluten-free bread; WGFB = whole-grain gluten-Free bread; ^a^ = values followed by a different superscript letter in each column are significantly different (ANOVA; Tukey’s test; *p* < 0.05). The mean value of iAUC for standard bread (not gluten-free) is 577.38 ± 59.97. Values with a superscript letter and * showed significant differences in chemical composition among different lots of the same GFB samples.

**Table 3:** Difference percentages between mean values from chemical analysis and labels’ information of the samples (per 100 g).

Sample	Carbohydrates		Difference %	Protein		Difference %	Lipids		Difference %	Dietary Fiber		Difference %
	(g)			(g)			(g)			(g)		
	Analyzed Value ^1^	Label Value		Analyzed Value ^1^	Label Value		Analyzed Value ^1^	Label Value		Analyzed Value ^1^	Label Value	
GFB 1	32.32	38	−17.57%	4.34	3.2	+26.27% *	1.75	4	−128.57% *	17.21	10.4	+39.57% *
GFB 2	48.34	46	+4.84%	2.22	4.2	−89.19% *	4.36	5.6	−28.44% *	7.74	1.2	+84.50% *
GFB 3	41.79	56	−34.00% *	5.09	1.6	+68.57% *	2	6.4	−220.00% *	10.13	1.4	+86.18% *
GFB 4	52.56	42	+20.09% *	5.11	2.84	+44.42% *	2.09	5.7	−172.73% *	6.85	3	+56.20% *
GFB 5	48.35	42	+13.13%	8.34	5.7	+31.65% *	3.9	5.7	−46.15% *	7.69	2	+73.99% *
GFB 6	50.65	58	−14.51%	5.52	2.84	+48.55% *	2.28	5.7	−150.00% *	8.8	2	+77.27% *
GFB 7	48.64	40	+17.76%	6.72	2.84	+57.74% *	2.68	2.84	−5.97%	6.99	2	+71.39% *
GFB 8	48.57	44	+9.41%	5.3	8.56	−61.51% *	2.03	5.64	−177.83% *	7.64	2	+73.82% *
WGFB 1	39.66	40	+0.34% *	4.17	2.8	+32.85% *	0.5	2	−300.00% *	16.26	10	+38.50% *
WGFB 2	47.32	44	+7.02%	3.54	5	−41.24% *	3.9	8	−105.13% *	6.79	2.4	+64.65% *
WGFB 3	40.11	52	−29.64% *	5.87	2.4	+59.11% *	3.96	7.4	−86.87% *	12.56	3.8	+69.75% *
WGFB 4	47.95	42	+12.41%	6.75	5.7	+15.56%	2.03	5.7	−180.79% *	6.81	4	+41.26% *

^1^ Mean Values of triplicates of three different manufacturing lots (*n* = 9). Values presented with (*) showed >20% of variation.

## Supplementary Materials

The following are available online at https://www.mdpi.com/2072-6643/12/8/2234/s1, Figure S1: Mean glycemia values for all analyzed GFB, Figure S2: Word Cloud generated from ingredients’ frequency in GFB classified as high Glycemic Index (a) and medium Glycemic Index (b), Table S1: Mean glycemia values in Mmol/L for GFB samples.
