# Supplementary material for: Chemical Composition and Glycemic Index of Gluten-Free Bread Commercialized in Brazil
Source: Nutrients. 2020 Jul 27;12(8):2234. doi: 10.3390/nu12082234 (PMC7468724; doi:10.3390/nu12082234)
Supplement: Supplementary file 1 [file nutrients-12-02234-s001.pdf]

Table S1. Mean glycemia values in Mmol/L for GFB samples.

Time (minutes)	White Bread (Standard)	GFB 1	GFB 2	GFB 3	GFB 4	GFB 5	GFB 6	GFB 7	GFB 8	WGFB 1	WGFB 2	WGFB 3	WGFB 4
0	4.32	4.82	4.41	4.34	5.29	5.21	4.39	4.39	4.39	5.01	4.41	4.60	4.25
15	4.79	5.13	5.01	5.067	5.48	5.41	5.36	5.29	5.29	5.30	4.69	4.59	5.24
30	5.46	5.38	5.13	5.81	6.08	5.90	5.88	5.75	5.74	5.48	4.99	5.142	5.67
45	5.55	5.15	4.94	6.16	7.03	6.85	6.67	6.57	6.57	5.54	4.95	5.66	6.34
60	6.15	5.35	5.19	6.58	6.82	6.69	6.81	6.74	6.74	5.42	5.19	5.94	6.61
90	5.46	4.98	4.89	5.56	6.06	5.89	6.14	5.76	5.76	5.08	4.95	5.63	5.99
120	4.76	5.28	5.11	5.24	5.48	5.39	5.45	5.25	5.25	4.89	4.89	4.94	5.25

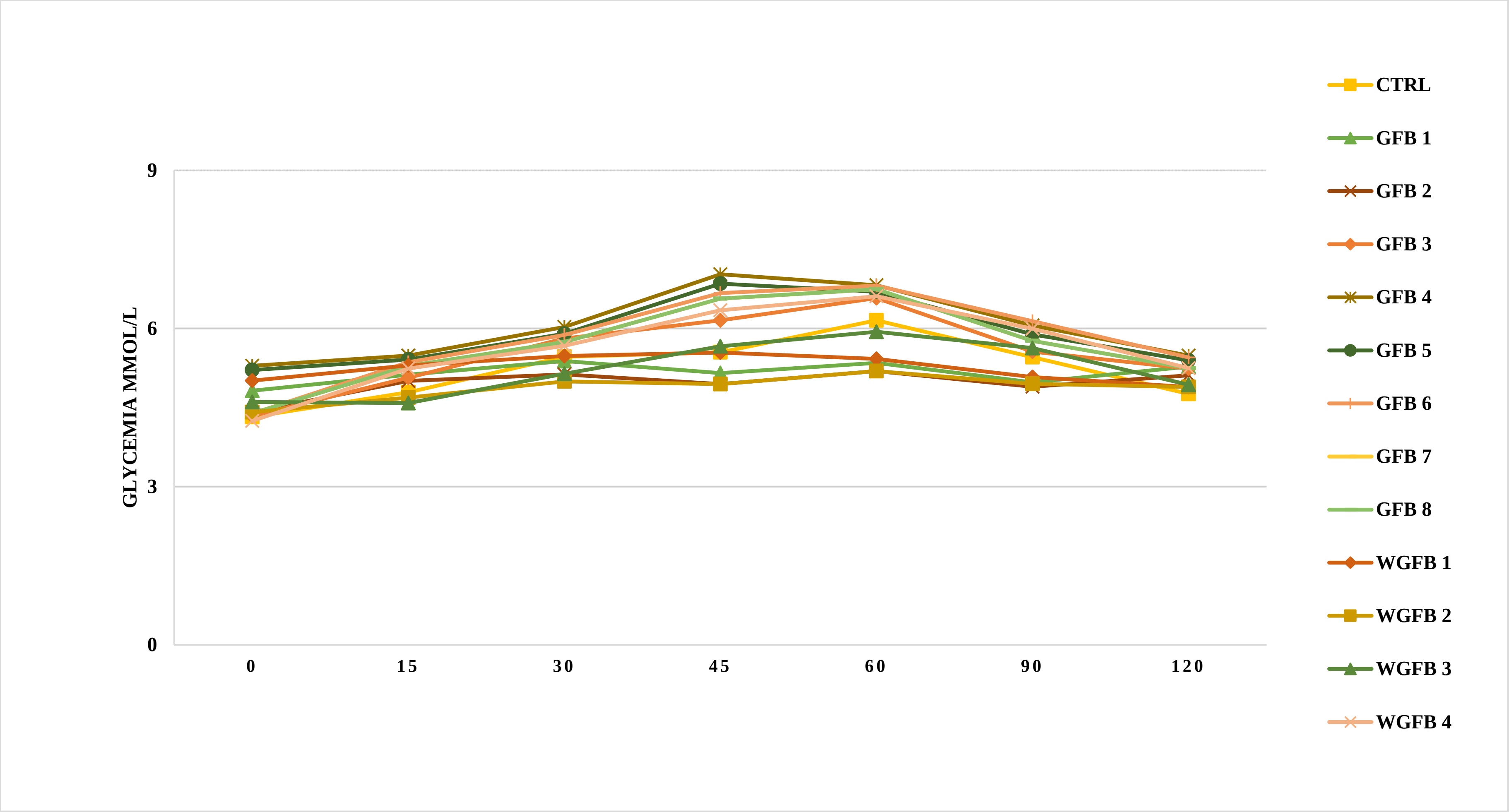

Figure S1. Mean glycemia values for all analyzed GFB

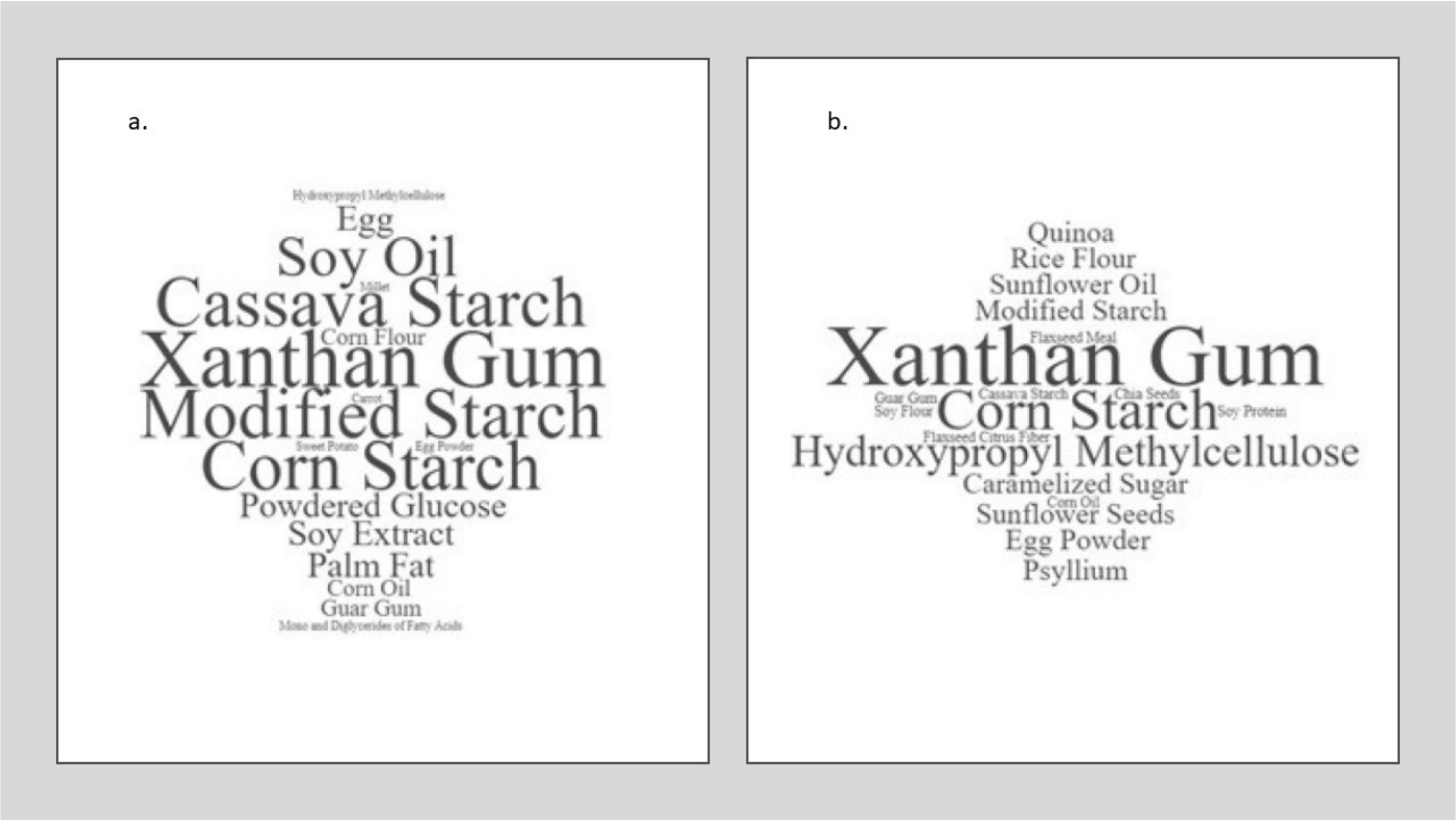

Figure S2. Word Cloud generated from ingredients' frequency in GFB classified as high Glycemic Index (**a**) and medium Glycemic Index (**b**).
